# Diphtheria in the former Soviet Union: reemergence of a pandemic disease.

**DOI:** 10.3201/eid0404.980404

**Published:** 1998

**Authors:** C R Vitek, M Wharton

**Affiliations:** Centers for Disease Control and Prevention, Atlanta, Georgia 30333, USA. cxv3@cdc.gov

## Abstract

The massive reemergence of diphtheria in the Newly Independent States of the former Soviet Union marked the first large-scale diphtheria epidemic in industrialized countries in 3 decades. Factors contributing to the epidemic included a large population of susceptible adults; decreased childhood immunization, which compromised what had been a well-established childhood vaccination program; suboptimal socioeconomic conditions; and high population movement. The role of a change in the predominant circulating strains of Corynebacterium diphtheriae in this epidemic remains uncertain. Massive, well-coordinated international assistance and unprecedented efforts to vaccinate adults were needed to control the epidemic.


### Diphtheria in the Former Soviet Union: Reemergence of a Pandemic Disease

**Charles R. Vitek and Melinda Wharton**
Centers for Disease Control and Prevention, Atlanta, Georgia, USA

The massive reemergence of diphtheria in the Newly Independent States of the former Soviet Union marked the first large-scale diphtheria epidemic in industrialized countries in 3 decades. Factors contributing to the epidemic included a large population of susceptible adults; decreased childhood immunization, which compromised what had been a well-established childhood vaccination program; suboptimal socioeconomic conditions; and high population movement. The role of a change in the predominant circulating strains of *Corynebacterium diphtheriae* in this epidemic remains uncertain. Massive, well-coordinated international assistance and unprecedented efforts to vaccinate adults were needed to control the epidemic.

In the 1990s, a massive epidemic throughout the Newly Independent States of the former Soviet Union marked the reemergence of epidemic diphtheria in industrialized countries. Diphtheria had been well controlled in the Soviet Union for more than 2 decades after universal childhood immunization was initiated in the late 1950s (Figure 1). Although all of the Newly Independent States were affected, three quarters of the more than 140,000 cases (Table 1) and two thirds of the more than 4,000 deaths reported since 1990 (1-3) were reported by the Russian Federation.

Massive efforts to vaccinate both children and adults are bringing the epidemic under control; in 1996, 20,215 cases were reported, a 60% decrease from the 50,425 cases reported in 1995 (4), and, in 1997, 6,932 cases were reported as of February 1998 (World Health Organization [WHO], unpub. data). The European Regional Office of WHO considers the outbreak nearly under control in Armenia, Azerbaijan, Belarus, Estonia, Latvia, Lithuania, Moldova, the Russian Federation, Turkmenistan, and Uzbekistan; in the five remaining republics, control is improving, but continuing efforts are needed to stabilize the situation.

This epidemic, primarily affecting adults in most Newly Independent States of the former Soviet Union, demonstrates that in a modern society diphtheria can still spread explosively and cause extensive illness and death. Intense international efforts have focused on aiding the affected countries and understanding the reasons for the epidemic. The study of this resurgence, especially as it relates to diphtheria resurgence in other industrialized countries, may elucidate the potential for the reemergence of other vaccine-preventable diseases.

Address for correspondence: Charles Vitek, Centers for Disease Control and Prevention, Mail Stop E61, Atlanta, GA 30333, USA; fax: 404-639-8616; e-mail: cxv3@cdc.gov.

### Epidemiology of Diphtheria

### Prevaccine Era

In the prevaccine era, diphtheria was a dreaded, highly endemic childhood disease found in temperate climates. Despite a gradual decline in deaths in most industrialized countries in the early 20th century, which was associated with improving living standards, diphtheria remained

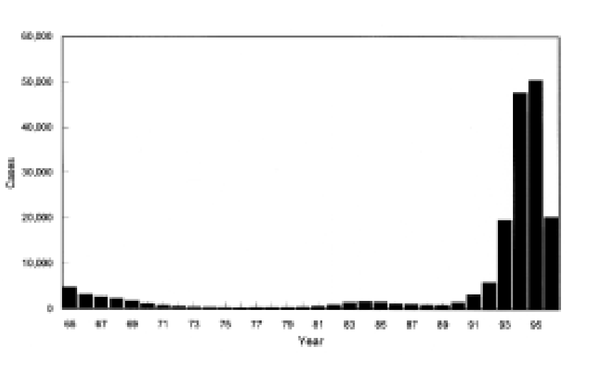

Figure 1. Reported diphtheria cases in the Soviet Union and the Newly Independent States, 1965–96.

Table 1. Diphtheria incidence in the Newly Independent States (NIS) of the former Soviet Union, 1991–1996

| Country | Population (millions) | Cases (per 100,000) 1991 | 1992 | 1993 | 1994 | 1995 | 1996 |
|---|---|---|---|---|---|---|---|
| Russia | 149.90 | 1,869 (1.25) | 3,897 (2.60) | 15,211 (10.15) | 39,582 (26.41) | 35,652 (23.78) | 13,604 (9.08) |
| Kazakstan | 17.46 | 30 (0.17) | 45 (0.26) | 82 (0.47) | 489 (2.80) | 1,105 (6.33) | 455 (2.61) |
| Kyrgyzstan | 4.7 | 10 (0.21) | 4 (0.09) | 6 (0.13) | 299 (6.36) | 693 (14.74) | 412 (8.77) |
| Tajikistan | 6.02 | 5 (0.08) | 14 (0.23) | 680 (11.30) | 1,912 (31.76) | 4,455 (74.00) | 1,464 (24.32) |
| Turkmenistan | 4.16 | 4 (0.10) | 22 (0.53) | 3 (0.07) | 60 (1.44) | 87 (2.09) | 80 (1.92) |
| Uzbekistan | 22.83 | 9 (0.04) | 29 (0.13) | 137 (0.60) | 224 (0.98) | 638 (2.79) | 160 (0.70) |
| Central Asia | 55.17 | 58 (0.11) | 114 (0.21) | 908 (1.65) | 2,984 (5.41) | 6,978 (12.65) | 2,571 (4.66) |
| Armenia | 3.74 | 0 (0.00) | 0 (0.00) | 1 (0.03) | 36 (0.96) | 29 (0.78) | 11 (0.29) |
| Azerbaijan | 7.46 | 36 (0.48) | 55 (0.74) | 141 (1.89) | 841 (11.27) | 883 (11.84) | 114 (1.53) |
| Georgia | 5.49 | 7 (0.13) | 3 (0.05) | 28 (0.51) | 294 (5.36) | 419 (7.63) | 346 (6.30) |
| Caucasus | 16.69 | 43 (0.26) | 58 (0.35) | 170 (1.02) | 1171 (7.02) | 1,331 (7.97) | 471 (2.82) |
| Ukraine | 52.47 | 1,103 (2.10) | 1,567 (2.99) | 2,987 (5.69) | 2,990 (5.70) | 5,280 (10.06) | 3,156 (6.01) |
| Moldova | 4.36 | 14 (0.32) | 22 (0.50) | 35 (0.80) | 376 (8.62) | 418 (9.59) | 97 (2.22) |
| Belarus | 10.33 | 26 (0.25) | 66 (0.64) | 120 (1.16) | 230 (2.23) | 322 (3.12) | 179 (1.73) |
| Western NIS | 67.16 | 1,143 (1.70) | 1,655 (2.46) | 3,142 (4.68) | 3,596 (5.35) | 6,020 (8.96) | 3,432 (5.11) |
| Estonia | 1.57 | 7 (0.45) | 3 (0.19) | 11 (0.70) | 7 (0.45) | 19 (1.21) | 14 (0.89) |
| Latvia | 2.65 | 5 (0.19) | 8 (0.30) | 12 (0.45) | 250 (9.43) | 369 (13.92) | 112 (4.23) |
| Lithuania | 3.77 | 1(0.03) | 9 (0.24) | 8 (0.21) | 38 (1.01) | 43 (1.14) | 11 (0.29) |
| Baltics | 7.99 | 13 (0.16) | 20 (0.25) | 31 (0.39) | 295 (3.69) | 431 (5.39) | 137 (1.71) |
| All NIS | 296.91 | 3,126 (1.05) | 5,744 (1.93) | 19,462 (6.55) | 47,628 (16.04) | 50,412 (16.98) | 20,215 (6.81) |

one of the leading causes of childhood death until widespread vaccination was implemented. In England and Wales, as recently as 1937 to 1938, diphtheria was second only to pneumonia among all causes of childhood death (5), with an annual death rate of 32 per 100,000 in children less than 15 years of age.

Most urban residents acquired immunity to diphtheria by the age of 15 years (6); only a minor portion of diphtheria cases were in adults. Only approximately 15% of children with immunity to diphtheria had had typical clinical diphtheria; the other 85% had milder symptoms or asymptomatic infections (6). Children of preschool and elementary school age had the highest attack rates. The age of school entry was associated with increased risk for disease (7). Peaks of endemic diphtheria in the fall (8) were often attributed to the opening of schools. Crowding and low socioeconomic conditions were other risk factors (9).

Superimposed on the high endemic disease rates was a rough periodicity of incidence with peaks every several years (5). Epidemic waves characterized by extremely high incidence and deaths were sporadic: Spain in the early 1600s (10), New England in the 1730s (10,11), and Western Europe from 1850 to 1890 (12,13). The factors governing diphtheria periodicity are not understood.

### Vaccine Era in Western Europe and the United States

In the United States, Canada, and many countries in Western Europe, the widespread use of diphtheria toxoid for childhood vaccination beginning in the 1930s and 1940s led to a rapid reduction in diphtheria incidence (14). However, in the 1930s, gradual rises in diphtheria incidence to 200 cases per 100,000 in the prewar period occurred in Germany and several other central European countries with partially implemented vaccination programs. The onset of World War II in 1939 and the occupation by German troops of many Western European countries led to the last diphtheria pandemic in Western industrialized countries. Although diphtheria incidence had been very low before the war, Holland, Denmark, and Norway had severe epidemics following occupation by German soldiers. Newly developed biotyping methods confirmed that endemic disease in prewar Germany was associated with strains of gravis biotype and that epidemics in occupied countries were associated with the introduction of gravis strains (15-17).

Data from a World War II epidemic in Halifax, Nova Scotia, suggest the high epidemic potential of these strains (18). Diphtheria was endemic in Halifax (30 to 80 cases per year), primarily among children in poor neighborhoods. Although not all isolates were biotyped, mitis strains seemed to be predominant. Gravis organisms, presumably related to the strains introduced into Norway by German troops, were introduced into Nova Scotia in September 1940 by Norwegian sailors with diphtheria, leading to an outbreak of 649 cases by July 1941. All biotyped strains were gravis. At first, secondary cases occurred predominantly among school-aged children; following cases were among adults, especially women. A study of more than 1,000 school children in February 1941 found gravis carriage rates of up to 30% in schools in poor neighborhoods. An apparently limited introduction of new strains resulted in an epidemic while circulation of the previously endemic strains had not.

Diphtheria incidence continued to decline steadily throughout the vaccine era in the United States (Figure 2) and (after the immediate postwar period) in Western Europe. Cases of clinical diphtheria have become extremely uncommon; several European countries have not reported a case of diphtheria in more than 20 years (12,19). Residual indigenous cases have been concentrated among incompletely vaccinated or unvaccinated persons of low socioeconomic status. In the United States, the decline was interrupted in the late 1960s by a small resurgence of diphtheria lasting until 1975 (20). Until this time, the predominant biotype in the United States, especially in the Southeast, was mitis, although gravis outbreaks were common in the West. Intermedius cases were uncommon, and outbreaks were rare (21). Between 1969 and 1975, outbreaks caused by intermedius strains were reported in economically depressed populations in Chicago (22), San Antonio (23), Phoenix (24), the Navajo reservation in Arizona and New Mexico (25), Seattle (26), and communities in eastern Washington State (Centers for Disease Control and Prevention [CDC], unpub. data). Since 1975, cases of respiratory diphtheria and isolation of toxigenic strains of *Corynebacterium diphtheriae* have been extremely rare in the United States (19,27).

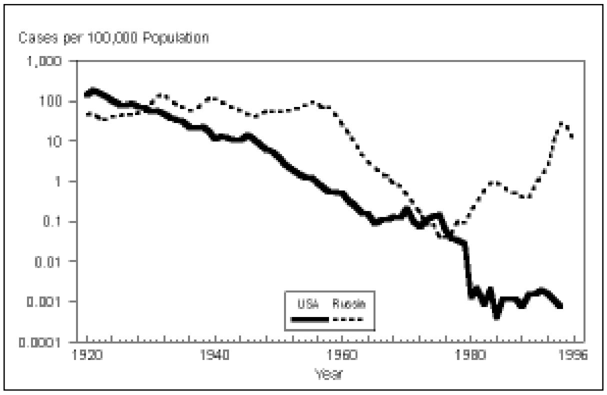

Figure 2. Diphtheria Incidence—United States and Russian Federation, 1920–1996.

### Prevaccine and Early Vaccine Era in the Soviet Union

Diphtheria incidence in Russia was high throughout the first half of the 20th century (Figure 2); more than 750,000 cases were reported in Russia alone in the 1950s. Although immunization against diphtheria began in some areas of the Soviet Union as early as the 1920s, it was only in 1958 that universal childhood immunization began throughout the Soviet Union (28). By 1963, the incidence in the Soviet Union had decreased 15-fold compared with 1958 and elimination was thought attainable. From 1965 until 1980, the Soviet childhood vaccination schedule mandated five doses of high antigenic content diphtheria vaccine by school entry and a school booster (Table 2). Diphtheria incidence levels continued to drop and in the mid-1970s approximated levels in the United States; only 199 cases (0.08 cases per 100,000) were reported in the Soviet Union in 1975 and only 198 cases in 1976. As in the prevaccine era, most cases were in children; in 1975 and 1976, 62% of the 109 cases in the Russian Federation were in children under 15 years of age (N.M. Maksimova, pers. comm.). During this period, diphtheria incidence was higher in the Central Asian republics (14).

### Resurgence and Stabilization of Diphtheria, 1977-1989

In 1977, diphtheria started to gradually make a comeback in the Soviet Union; incidence peaked in 1984 (1,609 cases, 0.9 per 100,000 population). The cases were concentrated in Russia, especially Central European Russia and the Russian Far East (29,30). In the Russian Federation, the resurgence was associated with a change in the predominant circulating biotype—from gravis (75% of cases 1975 to 1976) to mitis (60% of cases 1977 to 1981, 80% of cases 1982 to 1986). The circulating gravis strains belonged to several phage types and serotypes (31).

Table 2. Diphtheria vaccination schedule (Soviet Union 1965–1991, Russian Federation 1991–97, Ministry of Health, Russian Federation)

| | Year[a] | | | | | |
|---|---|---|---|---|---|---|
| | | 1980 | | 1987 | | |
| Age | 1965 | Alternative A | Alternative B | Alternative A | Alternative B | 1994 |
| 3-18 mo (infancy) | DTP (3 doses) | DTP (3 doses) | DT or Td (2 doses) | DTP (3 doses) | DT or Td (2 doses) | DTP (3 doses) |
| 18-36 mo[b] | DTP | DTP | DT or Td | DTP | DT or Td | DTP |
| 6 yr | DTP | Td | | Td | | |
| 9 yr | | | | Td | | |
| 11-12 yr | | Td | | Td | | Td |
| 16 yr | | Td (after 1983) | | Td | | Td |

[a]Antigenic content of Russian-manufactured vaccine: DTP = 15 lf units diphtheria toxoid per dose; DT = 30 lf units diphtheria toxoid per dose; Td = 5 lf units diphtheria toxoid per dose; d = 5 lf units diphtheria toxoid per dose.
[b]To be given 12-18 months after last infancy dose.

In 1978, for the first time most cases occurred in adults and, although incidence rates rose in all age groups, the rates in adults rose more rapidly and to higher levels than in children. The proportion of cases associated with mitis strains was also lower in children, suggesting that children were initially relatively protected or not exposed during this epidemic (29,30). In response to the resurgence, Soviet public health authorities intensified diagnostic and case investigation efforts, called for widespread vaccination of adult occupational groups at high risk, and in some localities with multiple cases, called for widespread community adult immunization (30).

However, changes in the immunization schedule during this period encouraged less intensive vaccination of children. Use of an alternative schedule of fewer doses of lower antigenic content (adult formulation) vaccine was allowed beginning in 1980; in 1986, the school entry booster dose was dropped, lengthening to 7 years the interval between recommended childhood booster doses after a primary series (Table 2). In addition, an increasing number of conditions were considered temporary or permanent contraindications to childhood vaccination. At the same time, public support for childhood vaccination programs fell, at least in some areas, for several reasons. Many childhood vaccine-preventable diseases had low incidence rates. In addition, a vocal antiimmunization movement received favorable press coverage in an atmosphere of increased distrust of government during perestroika (1985 to 1991). Participants in focus groups conducted in 1996 on diphtheria vaccination vividly recalled these reports and said that the reports had made them more fearful of vaccinating themselves or their children (N. Keith, pers. comm.). Childhood vaccination rates declined during the 1980s, with coverage of infants with a primary diphtheria toxoid series dropping to 70% or below for most of the decade (14). Many children vaccinated in the Russian Federation received low antigen content vaccine as their primary series, especially in some areas such as Moscow and St. Petersburg. In 1989 in Russia, more than 25% (a higher proportion than in any other republic [32]) of vaccinated children received primarily adult formulation vaccine preparations lacking the pertussis component.

After 1984, diphtheria incidence gradually declined to 839 cases (0.3 per 100,000) in 1989, although remaining above the nadir reached in 1976 to 1977. Most cases were reported by the Russian Federation; although most were in adults, the proportion of cases in children rose gradually throughout the 1980s (Figure 3).

The military may have played an important role in the persistence and spread of diphtheria

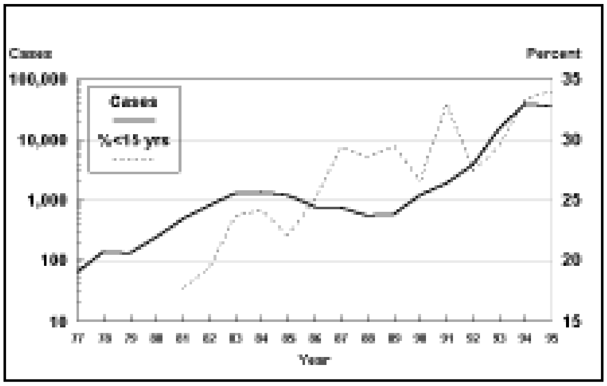

Figure 3. Reported cases and proportion of cases among children <15 years, Russian Federation. Data provided by the Russian Ministry of Health.

during the 1980s. Military service remained universal, and recruits were not routinely vaccinated against diphtheria until 1990. The source of one prolonged Russian outbreak (Kovrov District, Vladimir Oblast, 1982 to 1987) was diphtheria cases among a unit of military recruits from Central Asia that spread into the civilian population through social functions (V.A. Grigorevna, pers. comm.). Investigations in military units in various parts of Russia between 1983 and 1987 found carrier rates of toxigenic *C. diphtheriae* of up to 5.0% (33). Although cases in the military were (and still are) not reported to the Ministry of Health or included in the reported incidence data, some published data exist. An outbreak in Kzyl-Orda, Kazakstan, in 1988 began among the military but spread to the civilian population, causing 58 cases (34). From 1987 to 1990, most adult diphtheria patients seen at the Botkin Infectious Disease Hospital, one of two treatment units for diphtheria in Moscow, were members of the military (35). On the basis of published data, between 1990 and 1992 the minimum incidence of diphtheria in the military was 21 cases per 100,000 service members (36), six times higher than that of the civilian population (37). This incidence ratio was even more disproportionate in the late 1980s (32,36).

Beginning in 1986, gravis strains accounted for an increasing proportion of isolates, and in 1989, accounted for 52% of reported cases. One hundred and fifty-six diphtheria strains from various sites in Russia from 1985 to 1994 were analyzed by multilocus enzyme electrophoresis (MEE) and ribotyping. Although the mitis strains predominant in the 1980s were of multiple electrophoretic and ribotypes, most of the gravis strains from 1986 to 1989 belonged to a group of strains closely related by both MEE and ribotyping; this epidemic clone became predominant overall in the 1990s epidemic in Russia (38).

### Epidemic Diphtheria, 1990 to 1996

### Spread from the Center: 1990 to 1992

In 1990, 1,431 cases were reported in the Soviet Union, a 70% increase over 1989 (32). Cases were heavily concentrated in the Russian Federation (1,211 cases), especially Moscow City and Oblast (541 cases combined), and the three Pacific Coast oblasts of Khabarovsk, Primorye Krai, and Sakhalin Island (109 cases). In 1991, 3,126 cases were reported from the now Newly Independent States; epidemic diphtheria had reached St. Petersburg City and Oblast (246 cases) and in the Ukraine, Kiev (372 cases), Kharkov (129 cases), and Lvov (190 cases). Further spread within Russia, the Ukraine, and Belarus accounted for most of the 5,744 cases reported in 1992 (14).

### Explosion and Spread: 1993 to 1994

In 1993, the number of reported diphtheria cases surged to 19,462; epidemic diphtheria became established throughout urban Russia, the Ukraine, and Belarus. Russia alone reported 15,211 cases, an increase of 290% from 1992, with more cases reported in each succeeding month. For the first time in the epidemic, a pronounced seasonal incidence was seen (Figure 4), and the incidence rate in children exceeded that in adults by 60%. Elsewhere, Azerbaijan reported 141 cases and in the aftermath of a civil war, an epidemic sprang up in Tajikistan (680 cases) that spilled over into neighboring areas of densely populated Uzbekistan (137 cases). Cases had also increased in other Newly Independent States, although the incidence was still less than 1 per 100,000.

In 1994, epidemic diphtheria was reported from all states except Estonia, where most of the adult population had been vaccinated in 1985 to 1987. Russia had 39,582 (83%) of the 50,412 cases reported by the Newly Independent States. In Central Asia, areas of high incidence included regions adjacent to Russia in Kazakstan and regions adjacent to Tajikistan in both Uzbekistan and Kyrgyzstan, where the outbreak reportedly

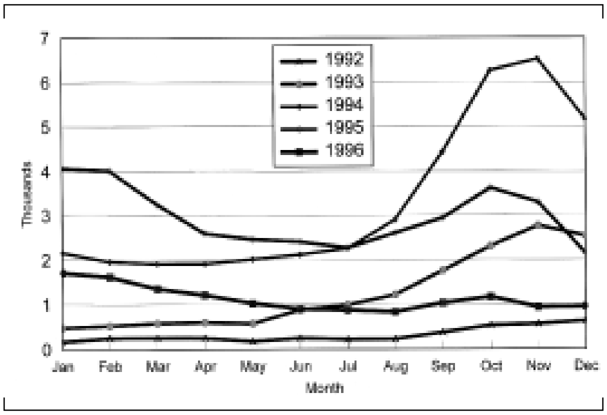

Figure 4. Diphtheria cases in the Russian Federation, 1992–96. 1994 = 39,582; 1995 = 35,652 (-10%); 1996 = 13,604 (-62%).

began with eight cases in a refugee family from Tajikistan (39).

In Russia, the Ukraine, Belarus, and the Baltics, most cases occurred in adults (3). In Russia in 1993, two thirds of cases were in persons older than 14 years. The highest incidence rates were among school-aged children and adolescents (12.4 to 18.2 per 100,000) and in adults ages 40 to 49 years (16.7 per 100,000); 45% of all deaths and the highest death rate (1.3 per 100,000 population) were reported among persons 40 to 49 years of age. Incidence rates dropped sharply in persons above age 50 (2.8 per 100,000) (40). Similarly in the Ukraine, adolescents 15 to 19 years of age and adults 40 to 49 years of age had the highest incidence rates (41). In several areas, most reported adult diphtheria cases were in women; women accounted for 60% of adult cases in St. Petersburg in 1991 to 1992 (42) and for 64% in three Russian regions in 1994 to 1995 (43). In the three regions, incidence rates among women 20 to 49 years of age were 68% higher than the rates among men of that age.

Biotype gravis strains have been predominant in Russia in the 1990s. Molecular studies using ribotyping and MEE demonstrated emergence of an epidemic clone of closely related strains (38,44). In the Ukraine, Belarus, the Baltics, and northern Kazakstan, the predominant strains were gravis biotype; in Tajikistan, Uzbekistan, Kyrgyzstan, and southern Kazakstan, the mitis biotype strains predominated.

### Public Health Response and Control of the Epidemic: 1995 to 1996

Initial diphtheria control efforts were hindered by shortfalls in strategy and vaccine supply. In the early years of the epidemic, public health officials concentrated on improving childhood coverage rates and on vaccinating adults in occupational groups perceived at high risk; vaccination of all adults was not directed by Russian public health authorities until 1993. The resulting unprecedented demand for adult formulation vaccine was met with stepped-up Russian vaccine production during 1994 to 1995 when nearly 80 million doses of adult formulation vaccine was produced (compared with less than six million in 1992 [Russian Federation Ministry of Health, unpub. data]).

Implementation efforts focused on vaccinating adults at work sites, followed by intensified efforts including house-to-house visits, to reach and vaccinate nonworking adults. Similarly, to further raise childhood coverage, a shortened list of contraindications was adopted and the use of full-strength vaccine preparations in the primary series was increased. In October 1994, the school-entry booster dose was reinstituted.

By the end of 1995, considerably improved coverage in children in Russia was reported (93% coverage with primary series at 1 year reported compared with 68.7% in 1991). Adult coverage with one or more doses in the previous 10 years was estimated at 75%; between January 1993 and December 1995, 70 million adults were vaccinated in the Russian Federation (45). In 1995, the incidence (24 cases per 100,000 population) decreased 10% from 1994. In 1996, 13,604 cases (9 per 100,000) were reported, a further 62% decline (2,4). In 1997, a provisional total of 4,057 cases were reported to WHO (WHO, unpub. data).

Unlike Russia, other Newly Independent States of the former Soviet Union states were not producers of diphtheria vaccine. The disruption of vaccine supply and economic difficulties associated with the dissolution of the Soviet Union were reflected in sharp drops in childhood vaccination rates in the early 1990s in the Central Asian and Caucasian republics (3).3 In 1994 to 1995, WHO, United Nations Children's Fund (UNICEF), other agencies, and Newly Independent States' governments developed and adopted an epidemic control strategy that aimed at rapidly raising adult and childhood coverage through nationwide immunization campaigns. Subsequently, a massive international effort, involving governmental and nongovernmental organizations and United Nations agencies and coordinated by an oversight committee, the Interagency Immunization Coordinating Committee, proved successful in mobilizing resources, purchasing and delivering vaccine to the Newly Independent States, and providing technical assistance to implement the strategy (2,3).

In 1995 and 1996, the Newly Independent States raised adult and childhood coverage and began to control the epidemic. All states made widespread use of workplace vaccinations for adults and other intensified vaccination tactics; these efforts resulted in increased adult coverage. The strategy was implemented with highly successful national mass campaigns in Moldova, Tajikistan, Latvia, Lithuania, and Azerbaijan, which achieved high adult coverage and rapid steep declines in incidence. All countries also made efforts to increase childhood coverage and to limit contraindications and the use of lower antigen content vaccine; routine childhood coverage exceeded 90% in most countries (2). Most countries reinstated a school-entry revaccination, and many of the national mass vaccination campaigns focused on children and adolescents. In 1996, 6,611 diphtheria cases were reported in the Newly Independent States excluding Russia, a 55% decrease compared to 1995; in the first quarter of 1997, 885 cases were reported, a 57% decrease compared with the first quarter of 1996 (4). WHO has received provisional reports of 2,875 cases for the full year of 1997, although data are not complete for a few countries (WHO, unpub. data).

### Factors Contributing to the Diphtheria Epidemic in the Newly Independent States

The control of epidemic diphtheria by childhood vaccination has been one of the outstanding successes of medicine in this century. Most Western industrialized countries have nearly eliminated this disease; many developing countries have progressively increased vaccination coverage by introducing diphtheria toxoid into vaccination programs. Global diphtheria incidence declined approximately 70% between the mid-1970s and the early 1990s (14). The diphtheria epidemic in the Newly Independent States raised numerous concerns about the efficacy of diphtheria control programs and of the diphtheria vaccine itself. However, case-control studies in the Ukraine and in Moscow demonstrated that three or more doses of Russian-manufactured diphtheria toxoid was highly effective in preventing disease in children (46). The rapid decline in disease incidence with increased vaccination coverage among both adults and children provides strong evidence of the continued effectiveness of diphtheria vaccine.

Numerous factors appear to have contributed to the epidemic: 1) increased adult susceptibility, which is reflected in the age distribution of cases and deaths; 2) increased susceptibility of children; 3) a clone of closely related strains of *C. diphtheriae*, gravis biotype, associated with most of the cases in Russia, even though its role remains uncertain; 4) highly crowded urban populations and service in the military; 5) the breakup of the former Soviet Union, perhaps by disrupting vaccine supply to all countries other than Russia and initiating large-scale population movements throughout the Newly Independent States.

### Increased Adult Susceptibility

Arguably the most important factor for the diphtheria epidemic was the development of large populations of adults susceptible to the disease as a consequence of successful childhood vaccination programs. The decreased opportunity for naturally acquired immunity, along with the waning of vaccine-induced immunity in the absence of routine adult revaccination, has resulted in a high proportion of adults susceptible to diphtheria as documented by serologic studies in many countries (12). In the United States, a trend to increased diphtheria incidence in older age groups was noted before the near-total disappearance of diphtheria (47). In several developing countries that have conducted immunization programs for more than 10 years, recent small epidemics have shown a similar increase in the proportion of adult cases (12).

Adults born between the early 1940s and the late 1950s in the Russian Federation and some other Newly Independent States were at the highest risk for never having acquired immunity to diphtheria; during their childhood, diphtheria incidence was decreasing, but not all children were reached by newly implemented vaccination programs. The gap in immunity in this age group, observed in serologic studies (48,49), was reflected in the very high numbers of deaths and illnesses among persons 35 to 50 years of age. Adults with similar high susceptibility to diphtheria in the United States and Western Europe are likely to be older than susceptible adults in the former Soviet Union because of the earlier implementation of vaccination programs in the West. The only sizeable diphtheria outbreaks in the United States that involved predominantly adults occurred in the early 1970s in Arizona and Washington. At this time, the analogous cohort of U.S. adults born just before widespread immunization would have been approximately 30 to 50 years old. Points of maximal population susceptibility to epidemic adult diphtheria therefore may depend on the age distribution of adults susceptible to the disease and the frequency of high contact rates for these susceptible adults (e.g., military service for young adults, care of school-aged children for young and middle-aged adults, homelessness among young and middle-aged men).

A high rate of adult susceptibility to diphtheria is a necessary but not sufficient precondition for the development of epidemic diphtheria in adults. The United States and most other European countries have high rates of adult susceptibility but have not had sustained chains of transmission, despite documented importations. In Poland and Finland, which border on the Newly Independent States that had diphtheria epidemics, multiple documented importations since 1990 have led to only very limited secondary transmission; these countries have maintained very high levels of childhood coverage against diphtheria (50).

In the epidemic of the Newly Independent States, sustained transmission in adults may have been limited to certain focal groups. In addition to military units, high-level transmission between adults (clusters of multiple cases and high rates of carriage) was demonstrated in other adult groups characterized by crowding, low levels of hygiene, and high contact rates, such as the homeless and patients in neuropsychiatric hospitals (40,42); in routine work settings, clusters of cases were rare, and the carrier rates among adult contacts of cases were very low.

### Increased Childhood Susceptibility

By 1990, on-time coverage of infants and young children had fallen because of changes in childhood vaccination recommendations and practices and increased population skepticism regarding vaccination, which was greatly exacerbated by an active antivaccination movement. Vaccination was frequently delayed and temporary contraindications to vaccination were extremely common. In addition, many children were considered to have permanent contraindications to vaccination, large numbers of children received lower antigen content vaccines in the primary series, and the interval between childhood booster doses was lengthened. The lowered coverage with pertussis-containing vaccine has been linked to a resurgence of pertussis in the Russian Federation (51). Some data exist on the direct impact of these changes on diphtheria incidence; in Russia and the Ukraine, incidence was higher among unvaccinated than vaccinated children, and lack of vaccination was a strong risk factor for severe disease (40). A case-control study after the reinstitution of the school entry booster dose found an interval of greater than 5 years since the last dose to be a strong risk factor for disease (52). No data regarding the effect of the increased use of lower antigen content vaccine are available.

Although since the mid-1970s most of the diphtheria cases in the Newly Independent States have been in adults, throughout the 1980s and early 1990s the proportion of diphtheria cases in children increased during the period of worsening overall control of diphtheria in all age groups (Figure 3). The increase in diphtheria cases among children suggests an important role for the susceptibility of children in the dissemination of diphtheria. In addition, most reported carriers in the Russian Federation were children in both the 1980s (31) and 1990s (Russian Federation Ministry of Health, unpub. data). Clusters of multiple cases in schools and within families were prominent features in this epidemic, as in previous ones (40). Some data suggest that a proportion of the disease in adults may represent 'sentinel' events and may have been transmitted from ill or asymptomatic children; these data include the much higher disease incidence among women compared with men (despite no evidence of lower serologic immunity [50,53]) and the large proportion of adult cases linked with multiple asymptomatic child carriers (CDC and Russian Federation Ministry of Health, unpub. data). In this respect, epidemic diphtheria in the era of adult susceptibility may resemble epidemic influenza, in which studies suggest that schoolchildren are a very important population in transmission and spread, although the bulk of severe disease occurs in their adult contacts (54).

### Change in Biotype or Epidemic Clone

Changes in the circulating strains of *C. diphtheriae* could be responsible for the cyclicity and episodic epidemic waves associated with diphtheria incidence in the prevaccine era. The outbreaks in Western Europe during World War II, in the United States in the 1970s, and in the Soviet Union/Newly Independent States in the mid-1970s and the late 1980s were associated with the emergence of a new biotype. Host factors (such as antimicrobial immunity) could contribute to the epidemic potential of a newly introduced strain, but microbial factors cannot be excluded. However, the role of antibacterial immunity in preventing infection with *C. diphtheriae* has not been studied since the 1930s, and no microbial factors have been identified that distinguish epidemic from nonepidemic strains. The molecular characterization of an epidemic clone of gravis strains associated with the current epidemic in most parts of Russia supports a role for a change in the agent in the development of the epidemic in the Newly Independent States of the former Soviet Union; however, other countries of the Newly Independent States have had epidemic diphtheria linked to strains of both mitis and gravis biotypes.

Although the source of the epidemic strains in Russia is unclear, persistent foci of diphtheria in Russia are a possible source. Russia was never totally free of reported cases of diphtheria, and recent reports of persistent disease-endemic foci in the United States (55) and Canada (56) suggest that circulation of toxigenic strains of *C. diphtheriae* can occur for prolonged periods even in the absence of recognized clinical cases, at least in certain communities. Other suggested sources include persistent diphtheria foci in the Central Asian countries or importation by returning military units from the war in Afghanistan between 1979 and 1990.

### Soviet Economic Development and the Post-Soviet Economic Crisis

Soviet populations were highly urbanized, but because of economic growth lagging behind that in Western Europe and the United States, most city dwellers lived in crowded apartments. Many of the amenities conducive to decreasing transmission of bacteria were deficient or lacking, especially in public areas, including routine access to functioning faucets for hand washing. A case-control study of diphtheria cases in Georgia found an increased risk for diphtheria associated with sharing utensils, cups, glasses, or a bedroom and with decreased bathing (57).

The economic crisis of the post-Soviet period in all Newly Independent States may have worsened these living conditions and contributed to the epidemic. The crisis indisputably led to lowered childhood coverage in the Central Asian and Caucasian republics in the early 1990s and may have contributed to the high proportion of childhood diphtheria cases reported from many of these republics.

### Militarization

The Soviet Union was the most extremely militarized large country in the world, with 1.4% of its population armed (58). The high level of militarization and the lack of routine immunization of recruits resulted in bringing large numbers of susceptible adults together from all parts of the immense country under conditions of crowding and suboptimal hygiene. These adverse conditions may have played a role in the development of the epidemic as suggested by the high diphtheria incidence in the military early in the 1990s.

### Increased Travel and Mass Population Movement

Other major changes after the breakup of the Soviet Union in 1991 included loosening of controls on movement within countries and increasing movements of populations between the Newly Independent States. During the Soviet era, movement was restricted by regulations and housing shortages. The success of control measures in the epidemic of the early 1980s may have been enhanced by slower dissemination of toxigenic strains due to movement restrictions. Similarly, while the epidemic clone was already established in the Newly Independent States by 1991, epidemic spread may have been facilitated by the mass movements of populations, primarily the repatriation of ethnic Russians from Central Asian and Caucasian countries and the flight of refugees from fighting in Georgia, Armenia, Azerbaijan, and Tajikistan.

### Factors in Successful Epidemic Control

The epidemic was controlled by vaccination efforts that achieved very high childhood coverage and unprecedented coverage in adults. The control strategy was developed and refined on the basis of epidemiologic analyses of disease incidence and population immunity. Implementation of the strategy for all of the Newly Independent States, except Russia, required massive international assistance; the instrumental role of the Interagency Immunization Coordinating Committee in successfully coordinating the multiple partners in this effort should serve as a model for future international public health emergencies. Finally, an effective, although underfunded, system of primary health care and public health centers had functioned throughout the Soviet Union for decades. The health workers of this system played a critical role in rapidly implementing the control measures once adequate strategies and material resources were identified.

### Lessons for Other Potential Reemerging Diseases

The epidemic in the Newly Independent States was unexpected; however, many of the factors apparently contributing to the epidemic coincide with factors important in the emergence of infectious diseases (Table 3) (59) and are connected with other diseases and other countries. An increased susceptibility in adults to childhood diseases is a predictable consequence of successful childhood vaccination programs with vaccines that produce less than lifelong immunity; this type of susceptibility has been suggested as one factor contributing to the current increase in pertussis in the United States. Although extreme loss of confidence in immunization may not occur often in other countries, an increasing population resistance to childhood vaccination as a result of adverse publicity and a diminished perception of risk occurs commonly (60) and has contributed to outbreaks of pertussis in England and Sweden (61,62). The role of changes in etiologic agents contributing to emergence is under study for many diseases. A recent outbreak of pertussis in the Netherlands may be due to a change in the predominant circulating strains resulting in decreased vaccine efficacy in children (63). Rapid urbanization with large segments of the population living under suboptimal hygienic conditions is characteristic of rapidly industrializing nations, and mass population movements are regular accompaniments of sociopolitical instability. The reemergence of diphtheria in the Newly Independent States of the former Soviet Union demonstrates the continued threat of this disease and of other infectious agents that may exploit similar social and political vulnerabilities.

Table 3. Factors influencing the emergence of diphtheria, Newly Independent States (NIS), 1990–1996.

| |
|---|
| Technology and industry |
| Population of susceptible adults |
| Human demographics and behavior |
| Population resistance to vaccinating children |
| Changes in childhood vaccination schedule |
| High levels of militarization |
| Decreased social controls, increased travel |
| Microbial adaptation and change |
| Change in biotype or emergence of epidemic clones |
| Economic development and land use |
| Highly crowded and intense urbanization, substandard housing |
| Breakdown of public health measures |
| Decreased immunization in Central Asia and Caucasus due to breakup of Soviet Union |
| International travel and land use |
| Repatriation of Russian population from republics |
| Refugees from Tajikistan, refugees in Georgia |

Dr. Vitek is a medical epidemiologist in the National Immunization Program, CDC. His research focuses on diphtheria and pertussis; he has worked extensively in the Russian Federation and Kazakstan.

Dr. Wharton is chief, Child Vaccine Preventable Diseases Branch, National Immunization Program, CDC. Dr. Wharton's research focuses on the epidemiology of vaccine-preventable diseases, especially pertussis, varicella, mumps, and diphtheria.

### References

1. Dittmann S. Epidemic diphtheria in the Newly Independent States of the former USSR—situation and lessons learned. Biologicals 1997;25:179-86.
2. Centers for Disease Control and Prevention. Update: diphtheria epidemic—New Independent States of the former Soviet Union, January 1995-March 1996. MMWR Morb Mortal Wkly Rep 1996;45:693-7.
3. Hardy I, Dittmann S, Sutter R. Current situation and control strategies for resurgence of diphtheria in newly independent states of the former Soviet Union. Lancet 1996;347:1739-44.
4. Dittmann S, Ciotti M. Epidemic diphtheria in the newly independent states: IICC support and current situation. In: Proceedings of the Sixth Meeting of the Interagency Immunization Coordinating Committee (IICC); 1997 May 29-30; Oslo, Norway. Geneva: WHO Regional Office for Europe; Report No.: CMDS070103/7.
5. Russell WT. The epidemiology of diphtheria during the last forty years. British Medical Research Council Special Report Series 1943;247:1-51.
6. Frost WH, Frobisher M, Van Volkenburgh VA, Levin ML. Diphtheria in Baltimore; a comparative study of morbidity, carrier prevalence and antitoxin immunity in 1921-1924 and 1933-1936. American Journal of Hygiene 1936;24:568-86.
7. Doull JA. Factors influencing selective distribution in diphtheria. Journal of Preventive Medicine 1930;4:371-404.
8. Dudley SF, May PM, O'Flynn JA. Active immunization against diphtheria. British Medical Research Council Special Report Series 1934;195:1-140.
9. Zingher A. Diphtheria preventive work in the public schools of New York City. Archives of Pediatrics 1921;38:336-59.
10. Holmes WH. Bacillary and Rickettsial infections: acute and chronic. New York: Macmillan Company; 1940. p. 289-305.

11. Caulfield E. A history of the terrible epidemic vulgarly called the throat distemper, as it occurred in His Majesty's New England colonies between 1735 and 1740. Yale J Biol Med 1939;11:219-72, 277-335.
12. Galazka AM, Robertson SE. Diphtheria: changing patterns in the developing world and the industrialized world. Eur J Epidemiol 1995;11:107-17.
13. Creighton C. A history of epidemics in Britain. New York: Barnes & Noble; 1965. p. 678-746.
14. Galazka A, Robertson S, Oblapenko G. Resurgence of diphtheria. Eur J Epidemiol 1995;11:95-105.
15. Stowman K. Diphtheria pandemic recedes. Bull World Health Organ 1947;1:60-7.
16. McLeod JW. A survey of the epidemiology of diphtheria in north-west Europe and North America in the period 1920-1946. Journal of Pathology and Bacteriology 1950;62:137-56.
17. Stuart G. A note on diphtheria incidence in certain European countries. BMJ 1945;2:613-5.
18. Wheeler S, Morton A. Epidemiological observations in the Halifax epidemic. Am J Public Health 1942;32:947-56.
19. Chen RT, Broome CV, Einstein RA, Weaver R, Tsai TF. Diphtheria in the United States, 1971-81. Am J Public Health 1985;75:1393-7.
20. Brooks GF, Bennett JV, Feldman RA. Diphtheria in the United States, 1959-1970. J Infect Dis 1974;129:172-8.
21. Doege TC, Levy PS, Heath CW. A diphtheria epidemic related to community immunization levels and the health problems of migrant workers. Public Health Rep 1963;78:151-60.
22. Kallick CA, Brooks GF, Dover AS, Brown MC, Brolnitsky O. A diphtheria outbreak in Chicago. Illinois Medical Journal 1970;137:505-12.
23. Marcuse EK, Grand MG. Epidemiology of diphtheria in San Antonio, Texas, 1970. JAMA 1973;224:305-10.
24. Diphtheria Surveillance Report, No. 12. Atlanta (GA): Centers for Disease Control, 1978; DHEW Pub. No. (CDC) 78-8087.
25. Center for Disease Control. Follow-up on diphtheria on a Navajo Indian reservation-Arizona, New Mexico. MMWR Morb Mortal Wkly Rep 1973;22:379-80.
26. Harnisch JP, Tronca E, Nolan CM, Turck M, Holmes KK. Diphtheria among alcoholic urban adults. Ann Intern Med 1989;111:71-82.
27. Bisgard K, Hardy I, Popovic T, Strebel PM, Wharton M, Chen RT, et al. Respiratory diphtheria in the United States, 1980-1995. Am J Public Health 1998;88:787-91.
28. Khazanov MI. On the problem of the elimination of diphtheria. Vestn Ross Akad Medi Nauk 1964;(8):13-20.
29. Sukhorukova NL, Tymchakovskaya IM, Gimpelevich SD, Markina SS. Epidemiology of diphtheria in the RSFSR under the conditions of mass immunization for many years. Zh Mikrobiol Epidemiol Immunobiol 1983;60:94-9.
30. Markina SS, Tymchakovskaya IM, Maksimova NM, Sukhorukova NL. The epidemic process of diphtheria in the RSFSR during the introduction of epidemiologic surveillance. Zh Mikrobiol, Epidemiol Immunobiol 1989;66:38-42.
31. Sukhorukova NL, Gempelevich SD, Favorova LA, Markina SS, Cherkasova VV. Results of conducting an epidemiological survey of diphtheria in 21 territories of the RSFSR. Zh Mikrobiol, Epidemiol Immunobiol 1983;60:95-8.
32. World Health Organization. Expanded programme on immunization: outbreak of diphtheria, USSR. Wkly Epidemiol Rec 1991;66;181-8.
33. Kolkov VF, Rusakova EV, Vasiveva VI, Alexandrova LI, Bezuglova MC. The condition of antidiphtheria immunity and carriage of toxigenic corynebacteria diphtheriae in organized collectives of adults. Voen Med Zh 1990;(11):42-4.
34. Narkevich MI, Onishchenko GG, Bolotovskij WM, Pichushkov AV, Lazikova GF, Boiko LS, et al. Status of immunoprophylaxis in the USSR and ways of its improvement. Zh Mikrobiol Epidemiol Immunobiol 1990;67:44-50.
35. Nikiforov VN, Turyanov MK, Belyaeva NM, Strakhov RV, Nikitina LA, Tashpulatov WA, et al. Clinical appearance, diagnosis, and treatment of diphtheria among adults. Ter Arkh 1995;67:16-8.
36. Lyashenko YI, Velichko MA. Special features of diagnosis and treatment of diphthera in military units and military treatment institutions. Voen Med Zh 1993;(10):37-40.
37. World Health Organization. Expanded programme on immunization: diphtheria outbreak in the Newly Independent States of the former USSR, 1990-94. Wkly Epidemiol Rec 1995;70;141-4.
38. Popovic T, Kombarova SY, Reeves MW, Nakao H, Mazurova IK, Wharton M, et al. Molecular epidemiology of diphtheria in Russia, 1985-1994. J Infect Dis 1996;174:1064-72.
39. Nuorti P, Kadyrova R, Firsova S, Rozkova L, Roure C, Dittman S, et al. Escalating epidemic of diphtheria, Kyrgyzstan 1994-1995. In: Abstracts of the 35th Interscience Conference on Antimicrobial Agents and Chemotherapy. 1995 Sep 17-20; San Francisco, California. Washington (DC): American Society for Microbiology. Abstract K171.
40. Markina SS, Maksimova NM, Bogatyreva EY, Kotova EA, Zhilina NY, Podunova LG, et al. Incidence of diphtheria in Russia in 1993. In: Monisov AA, Podunova LG, Tyasto AS, Emeljanov OV, Churchill RE, editors. The health of the population and the environment: Moscow: Russian Federation State Committee on Sanitary Epidemiologic Surveillance, 1994;(11):10-5.
41. World Health Organization. Expanded programme on immunization: diphtheria epidemic. Wkly Epidemiol Rec 1994;69:253-8.
42. Kurchanov V, Parkov O, Timopheeva E, Noscov F. Diphtheria epidemiology in St. Petersburg: epidemiology and control. In: Proceedings of Meeting on Diphtheria Epidemic in Europe; St. Petersburg; 1993 5-7 Jul. Lyon/Copenhagen: Foundation Marcel Merieux and WHO; 1994.

43. Vitek C, Bisgard K, Lushniak B, Lyerla R, Zhilina N. Diphtheria epidemic in the Russian Federation: evidence for a decline in incidence. In: Abstracts of the 36th Interscience Conference on Antimicrobial Agents and Chemotherapy. 1996 Sep 15-18; New Orleans, Louisiana. Washington (DC): American Society for Microbiology. Abstract G94.
44. De Zoysa A, Efstratiou A, George RC, Jahkola M, Vuopio-Varkila J, Deshevoi S, et al. Molecular epidemiology of *Corynebacterium diphtheriae* from northwestern Russia and surrounding countries studied by using ribotyping and pulsed-field gel electrophoresis. J Clin Microbiol 1995;33:1080-3.
45. World Health Organization. Evaluation of measures for poliomyelitis eradication and diphtheria control. Report on a WHO meeting, Berlin, Germany; 1996 Jun 3-5. Geneva: WHO Report No. EUR/ICP/CMDS 07 01 04.
46. Hardy I. Effectiveness of diphtheria vaccine: results of studies in Russia and Ukraine. In: Proceedings of Meeting on Diphtheria Epidemic in Europe, St. Petersburg, 1993 Jul 5-7. Lyon/Copenhagen: Foundation Marcel Merieux and WHO; 1994.
47. Naiditch M, Bower A. A study of 1,433 cases observed during a ten-year period at the Los Angeles County Hospital. Am J Med 1954;17:229-45.
48. Maksimova NM, Sukhorukova NL, Kostyuchenko AV, Michailova NB, Demina DI, Shestakov EN, et al. Specific prophylaxis of diphtheria in adults in the focus of diphtheria infection. Zh Mikrobiol Epidemiol Immunobiol 1987;64:36-40.
49. Hardy I, Kozlova I, Tchoudnaia L, Gluskevic T, Marievsky V, Sutter R, et al. Immunogenicity of Td vaccine in Ukrainian adults. In: Abstracts of the 35th Interscience Conference on Antimicrobial Agents and Chemotherapy 1995. 1995 Sep 17-20; San Francisco, California. Washington (DC): American Society for Microbiology. Abstract G25.
50. Galazka A, Tomaszunas-Blaszczyk J. Why do adults contract diphtheria? Eurosurveillance 1997;2:60-3.
51. Gangarosa E, Galazka A, Wolfe C, Phillips L, Gangarosa R, Miller E, et al. Impact of anti-vaccine movements on pertussis control: the untold story. Lancet 1998;351:356-61.
52. Vitek C, Brennan M, Gotway C, Bisgard K, Strebel P, Rhodes P, et al. Risk for diphtheria among children associated with increasing time since last booster vaccination. In: Abstracts of the 37th Interscience Conference on Antimicrobial Agents and Chemotherapy 1997. 1997 Sep 28-Oct1; Toronto, Canada. Washington (DC): American Society for Microbiology. Abstract G-9.
53. Jenum PA, Skogen V, Danilova E, Eskild A, Sjursen H. Immunity to diphtheria in northern Norway and northwestern Russia. Eur J Clin Microbiol Infect Dis 1995;14:794-8.
54. Glezen WP. Emerging infections: pandemic influenza. Epidemiol Rev 1996;18:64-76.
55. Centers for Disease Control and Prevention. Toxigenic *Corynebacterium diphtheriae*—Northern Plains Indian community. MMWR Morb Mortal Wkly Rep 1997;46:506-10.
56. Cahoon F, Brown S, Jamieson F. *Corynebacterium diphtheriae*—toxigenic isolations from northeastern Ontario. In: Abstracts of the 37th Interscience Conference on Antimicrobial Agents and Chemotherapy 1997. 1997 Sep 28-Oct1; Toronto, Canada. Washington (DC): American Society for Microbiology. Abstract K171.
57. Quick L, Hardy I, Sutter RW, Strebel P, Kobaidze K, Malakmadze N. Risk factors for transmission of diphtheria among 219 cases in the Republic of Georgia; 1995-1996. In: Abstracts of the 37th Interscience Conference on Antimicrobial Agents and Chemotherapy 1997. 1997 Sep 28-Oct1; Toronto, Canada. Washington (DC): American Society for Microbiology. Abstract K168.
58. Britannica Book of the Year 1991. Chicago: Encyclopedia Britannica Inc.; 1991.
59. Lederberg J, Shope RE, Oaks SC Jr, editors. Emerging infections: microbial threats to health in the United States. Washington (DC): National Academy Press; 1992.
60. Chen RT, Rastogi SC, Mullen JR, Hayes S, Cochi SL, Donlon JA, et al. The Vaccine Adverse Event Reporting System (VAERS). Vaccine 1994;12:542-50.
61. Effect of a low pertussis vaccination uptake on a large community. Report from the Swansea Research Unit of the Royal College of General Practitioners. BMJ 1981;282:23-6.
62. Romanus V, Jonsell R, Bergquist S. Pertussis in Sweden after the cessation of general immunization in 1979. Pediatr Infect Dis J; 1987;6:364-71.
63. De Melker HE, Conyn-van Spaendonck MA, Rumke HC, van Wijngaarden JK, Mooi FR, Schellekens JF. Pertussis in the Netherlands: an outbreak despite high levels of immunization with whole-cell vaccine. Emerg Infect Dis 1997;3:175-8.
